# Development of GABAergic Inputs Is Not Altered in Early Maturation of Adult Born Dentate Granule Neurons in Fragile X Mice

**DOI:** 10.1523/ENEURO.0137-18.2018

**Published:** 2018-12-03

**Authors:** Christine L. Remmers, Anis Contractor

**Affiliations:** 1 Department of Physiology, Feinberg School of Medicine, Northwestern University, Chicago, IL 60611; 2 Department of Neurobiology, Weinberg College of Arts and Sciences, Northwestern University, Evanston, IL 60208

**Keywords:** adult born neurons, fragile X, GABA, neurogenesis, synapse

## Abstract

Fragile X syndrome (FXS) is the most common form of inherited mental retardation and the most common known cause of autism. Loss of fragile X mental retardation protein (FMRP) in mice (*Fmr1* KO) leads to altered synaptic and circuit maturation in the hippocampus that is correlated with alterations in hippocampal-dependent behaviors. Previous studies have demonstrated that loss of FMRP increased the rate of proliferation of progenitor cells and altered their fate specification in adult *Fmr1* KO mice. While these studies clearly demonstrate a role for FMRP in adult neurogenesis in the hippocampus, it is not known whether the functional synaptic maturation and integration of adult-born dentate granule cells (abDGCs) into hippocampal circuits is affected in *Fmr1* KO mice. Here, we used retroviral labeling to birthdate abDGCs in *Fmr1* KO mice which allowed us to perform targeted patch clamp recording to measure the development of synaptic inputs to these neurons at precise time points after differentiation. The frequency and amplitude of spontaneous GABAergic events increased over the first three weeks after differentiation; however, this normal development of GABAergic synapses was not altered in *Fmr1* KO mice. Furthermore, the relatively depolarized GABA reversal potential (*E*_GABA_) in immature abDGCs was unaffected by loss of FMRP as was the development of dendritic arbor of the adult generated neurons. These studies systematically characterized the functional development of abDGCs during the first four weeks after differentiation and demonstrate that the maturation of GABAergic synaptic inputs to these neurons is not grossly affected by the loss of FMRP.

## Significance Statement

Loss of fragile X mental retardation protein (FMRP) causes Fragile X syndrome (FXS), a devastating neurodevelopmental disorder that causes multiple alterations in the development of synapses and neurons. Previous studies have described a role for FMRP in neurogenesis and hippocampal-dependent conditioning tasks linked to neurogenesis. This study systematically assessed the functional development of GABAergic inputs to adult born dentate granule cells (abDGCs) during the first four weeks after differentiation of adult neural stem cells in *Fmr1* KO mice.

## Introduction

Fragile X syndrome (FXS) is the most common known genetic cause of autism and intellectual disability. FXS is caused by the expansion of the CGG repeat in the 5’ UTR of the *Fmr1* gene, which leads to hypermethylation, transcriptional silencing, and loss of expression of the protein product, fragile X mental retardation protein (FMRP; Willemsen et al., 2011). FMRP is an RNA-binding protein that regulates a large number of mRNAs, many of which encode synaptic proteins. Dysregulated expression of synaptic proteins is thought to perturb synapse maturation and plasticity; however, the specific mechanisms underlying synaptic and cognitive deficits in FXS remain unclear. There are a growing number of studies demonstrating that FMRP plays important roles in stem cells, including adult neural stem cells in the neurogenic niche in the subgranular zone (SGZ) of the hippocampus (Li and Zhao, 2014). The importance of adult-born dentate granule cells (abDGCs) to hippocampal function, memory processes, and potentially to several neuropsychiatric disorders (Gonçalves et al., 2016) has raised the possibility that alterations in neural stem cell proliferation and maturation and integration of abDGCs contribute to the pathology of FXS. Proliferation of adult neural progenitors is enhanced in mice lacking FMRP (*Fmr1* KO; Luo et al., 2010; but also see Eadie et al., 2009), and fate specification is altered with fewer neural progenitors differentiating into neurons (Luo et al., 2010). In addition, it has been reported that reduced survival of adult-born neurons leads to an overall reduction in the number of DGCs in *Fmr1* KO mice (Lazarov et al., 2012). Therefore, the evidence suggests that the number of abDGCs is reduced in *Fmr1* KO mice and that FMRP ablation specifically in adult neural stem cells results in cell autonomous effects on proliferation, fate specification, and hippocampal-dependent behaviors (Guo et al., 2011). Despite these known disruptions in neurogenesis in *Fmr1* KO mice, an outstanding question is whether FMRP loss also affects how abDGCs mature and integrate into the hippocampal network after differentiation.

In many neuronal types, FMRP and related proteins play a role in neuronal development and synaptic function. There are alterations in the development of synapses particularly during early developmental critical periods in the cortex in *Fmr1* KO mice (Nimchinsky et al., 2001; Cruz-Martín et al., 2010; Harlow et al., 2010). In several instances, these morphologic and functional changes normalize during development (Bureau et al., 2008); however, phenotypes associated with these circuits persist in adult *Fmr1* KO mice (Arnett et al., 2014; He et al., 2017). abDGCs also undergo critical periods of development during the first few weeks after differentiation when they are undergoing synapse formation and dendritic remodeling (Bergami et al., 2015) and have elevated plasticity (Ge et al., 2007b). However, it remains unknown whether loss of FMRP results in disruptions in synapse formation on abDGCs during this early developmental postmitotic period when they are actively integrating into the hippocampal network.

GABA synapses are the first inputs to form onto abDGCs and these synapses are initially excitatory due to a depolarized GABA reversal potential (*E*_GABA_) that results from a relatively high intracellular chloride concentration in young neurons (Overstreet Wadiche et al., 2005; Ge et al., 2006). These earliest GABA inputs are important for survival, dendritic development, and subsequent formation and unsilencing of glutamatergic synapses (Ge et al., 2006; Chancey et al., 2013; Song et al., 2013; Alvarez et al., 2016). The distinct characteristics of immature abDGCs endow these cells with properties that create unique roles for them in information processing (Ge et al., 2007a). Ablation of adult neurogenesis leads to impaired trace and contextual fear conditioning and pattern separation, indicating that newly generated neurons play a distinct role in learning that cannot be replicated by mature or developmentally born DGCs (Shors et al., 2001; Clelland et al., 2009; Nakashiba et al., 2012). Interestingly, these same behaviors that have been shown to require intact adult hippocampal neurogenesis are impaired in the *Fmr1* KO. *Fmr1* KO mice have deficits in trace fear conditioning (Zhao et al., 2005), and this phenotype can be recapitulated by specific deletion of *Fmr1* in adult neural stem cells (Guo et al., 2011). Therefore, while it is known that there are reductions in the number of abDGCs and correlated changes in behavior, it remains to be determined whether loss of FMRP results in changes in synaptic formation and maturation in abDGCs, which would alter their integration and activity in the hippocampal circuit.

Given the critical role of GABA signaling in early postmitotic maturation of abDGCs (Ge et al., 2006), and also the known alterations in GABA signaling during early postnatal development (He et al., 2014; Nomura et al., 2017) and in juvenile *Fmr1* KO mice (Paluszkiewicz et al., 2011; Martin et al., 2014; Zhang et al., 2017), we investigated whether the time course of development of inhibition onto abDGCs is altered in *Fmr1* KO mice.

## Materials and Methods

### Animals

All procedures related to the care and treatment of animals were performed in accordance with the Northwestern University Animal Care Committee’s regulations. *Fmr1* KO mice (C57Bl/6) were maintained by breeding heterozygous females with WT or KO males. All experiments were performed blind to genotype in age-matched male littermates. Tail biopsies were used to perform *post hoc* genotyping of all mice used in the study.

### Retroviral birthdating

A replication incompetent retrovirus based on Moloney murine leukemia virus (MMLV) expressing RFP was prepared as described (Tashiro et al., 2006, 2015). Briefly, HEK-293 cells stably expressing GP2 were cotransfected with RFP and VSVG using Lipofectamine 2000. The media were collected from transfected cells 3 and 6 d after transfection, filtered, and centrifuged at 25,000 rpm to precipitate the virus. Eight- to 10-week-old *Fmr1* WT or KO males were anesthetized using ketamine/xylazine and 1 μl of virus was injected bilaterally into the SGZ of the dentate gyrus at a rate of ∼0.3 μl/min.

### Slice preparation and electrophysiology

We prepared 250-μm coronal slices at 14, 21, and 28 (±1) days postinjection (dpi). Slices were prepared using a Leica Vibratome in ice-cold high-sucrose artificial CSF (ACSF) containing the following: 85 mM NaCl, 2.5 mM KCl, 1.25 mM NaH_2_PO_4_, 25 mM NaHCO_3_, 25 mM glucose, 75 mM sucrose, 0.5 mM CaCl_2_, and 4 mM MgCl_2_, equilibrated with 95% O_2_ and 5% CO_2_ and including 10 μM DL-APV and 100 μM kynurenate. Slices were heated to 28°C in the same sucrose-ACSF, then the sucrose solution was gradually exchanged for recovery ACSF containing the following: 125 mM NaCl, 2.4 mM KCl, 1.2 mM NaH_2_PO_4_, 25 mM NaHCO_3_, 25 mM glucose, 1 mM CaCl_2_, 2 mM MgCl_2_, 0.01 mM dL-APV, and 0.1 mM kynurenic acid.

After a 60-min recovery, individual slices were transferred to a recording chamber and continuously perfused with oxygenated ACSF containing 2 mM CaCl_2_ and 1 mM MgCl_2_ at an elevated temperature of 32°C. The dentate gyrus was visually identified and targeted recordings were made from RFP-expressing dentate granule cells. Recording electrodes were manufactured from borosilicate glass pipettes and had tip resistances of 4–6 MΩ when filled with internal recording solution. For whole-cell recordings, internal recording solution contained the following: 95 mM CsF, 25 mM CsCl, 10 mM Cs-HEPES, 10 mM Cs-EGTA, 2 mM NaCl, 2 mM Mg-ATP, 10 mM QX-314, 5 mM TEA-Cl, and 5 mM 4-AP, pH adjusted to 7.3 with CsOH. Data were collected and analyzed using pClamp 10 software (Molecular Devices). Neurons were voltage-clamped at –70 mV to record spontaneous IPSCs (sIPSCs) and miniature IPSCs (mIPSCs), which were isolated by inclusion of D-APV (50 μM), CNQX (10 μM), and TTX (1 μM) for mIPSCs. MiniAnalysis (Synaptosoft) was used to analyze sIPSCs and mIPSCs. For perforated patch recordings, the pipette solution contained the following: 150 mM KCl and 100 mM HEPES, pH adjusted to 7.2 with Tris-OH. The pipette tip was filled with gramicidin-free KCl solution and then backfilled with solution containing gramicidin (100 μg/ml). GABAergic currents were evoked using a picospritzer to deliver a 50-ms puff of 10 μM GABA in the presence of 50 μM D-APV and 10 μM CNQX. Responses were recorded at holding potentials between –100 and 0 mV. The GABA reversal potential was calculated as the *x*-axis intercept of the best-fit line of the current−voltage plot.

### Two-photon laser scanning microscopy

Labeled abDGCs were patched in the whole cell configuration as described above with Alexa Fluor 488 dye (50 μM) added to the internal solution. Dye was allowed to perfuse through the cell for ∼20 min before image acquisition. Fluorescent images were acquired with picosecond pulsed excitation at 790 nM. Images of the soma and dendrites were acquired with 0.19-μm^2^ pixels with 10-μs pixel dwell time with 1.0-μm focal steps. Neuron Studio was used to create 3-D reconstructions of the dendrites and morphology analysis was performed in NeuronStudio (Wearne et al., 2005) and ImageJ.

### Data analysis

Data analysis was performed using Microsoft Excel and OriginPro 2017 software. mIPSC and sIPSCs were analyzed using MiniAnalysis (Synaptosoft). Decay kinetics of mIPSC events was measured as the time to decay from 90% to 37% of the peak amplitude on the falling phase of the response. Comparisons were made with a Mann–Whitney *U* test, unless otherwise indicated. Differences were considered significant when *p* < 0.05. Data are shown as mean ± SEM.

## Results

### Development of spontaneous GABA currents in abDGCs in *Fmr1* KO mice

**Figure 1.**
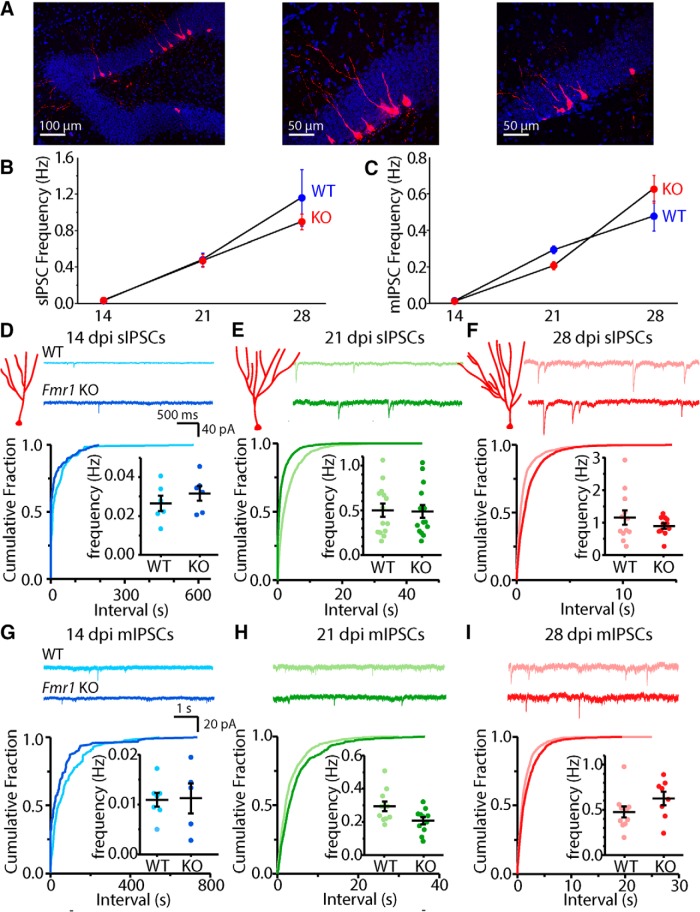
Frequency of sIPSCs and mIPSCs is not altered in abDGCs in *Fmr1* KO. ***A***, Representative images of 21 dpi abDGCs virally labeled with RFP. Average sIPSC (***B***) and mIPSC (***C***) frequencies across all time points measured in *Fmr1* WT (blue) and *Fmr1* KO mice (red). ***D***, Schematic of dendritic morphology of abDGCs and representative traces (top panel), cumulative distribution of interevent-interval and average frequency of each recorded neuron (inset) of sIPSCs (bottom panel) at 14 dpi, 21 dpi (***E***) and 28 dpi (***F***). ***G***, ***H***, ***I***, Representative traces, cumulative distribution of interevent-intervals and average frequency of mIPSCs in each recorded abDGC (inset) at 14, 21, and 28 dpi, respectively.

To identify and birthdate newborn DGCs, we injected a modified retrovirus expressing RFP into the SGZ of 8- to 10-week-old *Fmr1* KO mice and WT littermates (Tashiro et al., 2015). Retroviral injection clearly labeled neurons located in the SGZ of the dentate gyrus (Fig. 1*A*). Evoked IPSCs have been detected in abDGCs as early as 7 dpi (Ge et al., 2006) and in our recordings the earliest time point at which we consistently observed spontaneous inhibitory events was 14 dpi. Based on this we performed targeted patch-clamp recordings from RFP-expressing cells at 14, 21, and 28 dpi. We first measured the frequency of sIPSCs and mIPSCs at these time points. sIPSC frequency increased over time as abDGCs matured in both genotypes (Fig. 1*B*). Despite the known delays in maturation of properties of neurons in other cortical regions in *Fmr1* KO mice, we found that there was no difference in the sIPSC frequency at any postdifferentiation age of abDGC tested spanning this early period of development of these neurons (14 dpi WT: 0.028 ± 0.004 Hz; 14 dpi KO: 0.032 ± 0.004 Hz, *n* = 6/3 (cells, animals respectively), *p* = 0.49 Mann–Whitney *U* test; 21 dpi WT: 0.480 ± 0.074 Hz, *n* = 14/7, 21 dpi KO: 0.468 ± 0.070 Hz, *n* = 15/9, *p* = 0.95 Mann–Whitney *U* test; 28 dpi WT: 1.16 ± 0.031 Hz, *n* = 12, 4 KO: 0.90 ± 0.086 Hz, 11/6, *p* = 0.70 Mann–Whitney *U* test; Fig. 1*B*,*D–F*). In addition, we measured action potential independent spontaneous inhibitory events (mIPSCs) in abDGCs, which can be a good indicator of the number of inhibitory connections, or the release probability of those synapses. Again, we did not find a difference in the frequency of these events in comparisons between recordings in *Fmr1* WT and *Fmr1* KO mice at any of the ages tested (Fig. 1*C*,*G–I*). A comparison of the average mIPSC frequencies in each recording over time demonstrated an equivalent increase across this developmental period for abDGCs in both genotypes (Fig. 1*C*). Together, the lack of a difference in frequency of sIPSCs or mIPSCs during maturation of abDGCs indicates that there is no difference in the number of inhibitory synaptic connections in these neurons in the *Fmr1* KO mice during the first 4 weeks after differentiation.

**Figure 2.**
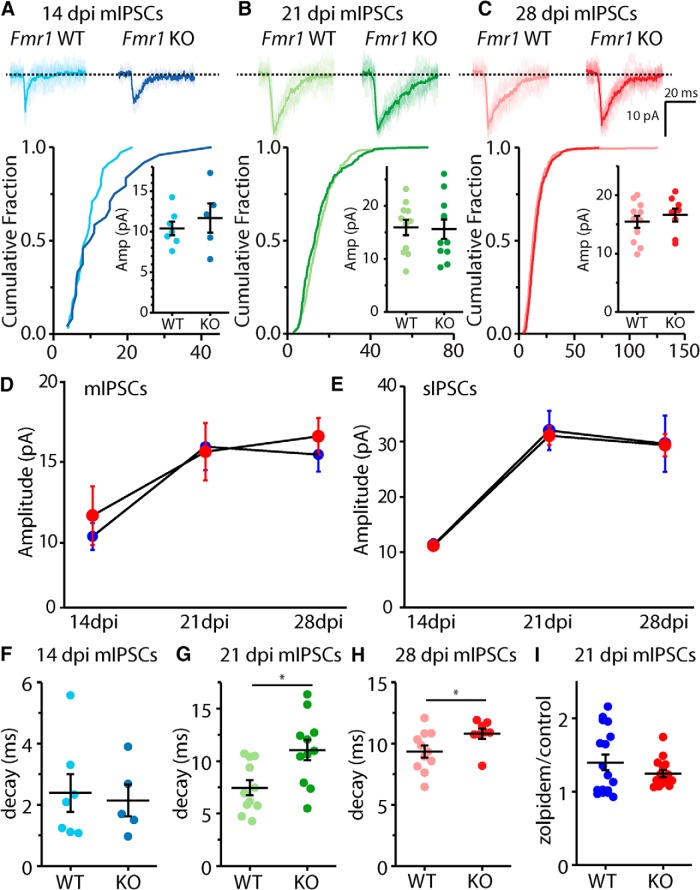
mIPSC amplitude is unaffected by loss of FMRP, but mIPSC decay is slower in abDGCs in *Fmr1* KO. ***A***, Representative traces of individual and averaged mIPSC events (top) and cumulative distribution and average amplitudes mIPSCs in each recording measured at 14 dpi, 21 dpi (***B***) and 28 dpi (***C***) abDGCs. Average mIPSC amplitude (***D***) and sIPSC amplitude (***E***) across all time points measured in *Fmr1* WT (blue) and KO (red). Average decay of mIPSCs in 14 dpi (***F***), 21 dpi (***G***), and 28 dpi (***H***) abDGCs. ***I***, Average mIPSC decay with zolpidem normalized to decay pre-zolpidem (*p < 0.05, Mann–Whitney *U* test).

The amplitude of spontaneous events, particularly mIPSCs, is an indicator of the strength of individual synapses and is expected to increase as abDGCs undergo maturation (Ge et al., 2006). Comparison of the mIPSC amplitudes at each of the days postdifferentiation spanning this period again did not reveal any difference between recordings in each of the genotypes (WT 14 dpi: 10.4 ± 0.827 pA, *n* = 7/3; KO 14 dpi: 11.7 ± 1.81 pA, n =,5/5, *p* = 0.64, Mann–Whitney *U* test; WT 21 dpi: 15.9 ± 1.45 pA, *n* = 12/5, KO 21 dpi: 15.6 ± 1.78 pA, *n* = 12/5, *p* = 1.00, Mann–Whitney *U* test; WT 28 dpi: 15.4 ± 1.04 pA, *n* = 11/4, KO 28 dpi: 16.6 ± 1.13 pA, *n* = 8/3, *p* = 0.49, Mann–Whitney *U* test; Fig. 2*A–C*). In addition, and consistent with no genotype-mediated changes in synaptic strength during the development of abDGCs in *Fmr1* KO mice, there were no differences in the amplitude of sIPSCs (Fig. 2*E*). In recordings of both mIPSCs and sIPSCs there was an equivalent increase of the average amplitude of events in older neurons in both genotypes (Fig. 2*D*,*E*). Interestingly, at 14 dpi, the average amplitude for both mIPSCs and sIPSCs was similar, suggesting that at this time point presynaptic inhibitory neurons make a single synaptic connection per axon. However, the amplitudes diverged at 21 dpi, with sIPSC amplitudes larger than those of mIPSCs, indicating that single presynaptic axons make multiple contacts onto abDGCs in these older neurons (Fig. 2*D*,*E*).

Measuring the decay of mIPSCs, we found that there was a significant increase in the decay time course as abDGCs matured in both genotypes that could be indicative of a change in the subunit composition of GABA_A_Rs (Overstreet Wadiche et al., 2005). At the youngest time measured mIPSC decay was not significantly different in *Fmr1* KO (WT 14 dpi: 2.39 ± 0.612 ms, *n* = 7/3; KO 14 dpi: 2.15 ± 0.517 ms, *n* = 5/4, *p* = 1.00, Mann–Whitney *U* test; Fig. 2*F*). However, at older ages of abDGCs, the decay of mIPSCs in *Fmr1* KO neurons was slower (WT 21 dpi: 7.45 ± 0.722 ms, *n* = 12/5; KO: 11.1 ± 0.987 ms, *n* = 12/5, *p* = 0.008, Mann–Whitney *U* test; WT 28 dpi: 9.36 ± 0.492 ms, *n* = 11/4; KO 28 dpi: 10.8 ± 0.410 ms, *n* = 8/3 cells, *p* = 0.041, Mann–Whitney *U* test; Fig. 2*G*,*H*). In addition, sIPSC decay was not significantly different in *Fmr1* KO (WT 14 dpi: 1.66 ± 0.275 ms, *n* = 6/3; KO 14 dpi: 1.57 ± 0.127 ms, *n* = 6/3; *p* = 0.7, Mann–Whitney *U* test; WT 21 dpi: 12.6 ± 1.19 ms, *n* = 14/8; KO 21 dpi: 16.0 ± 1.39 ms, *n* = 15/9, *p* = 0.08, Mann–Whitney *U* test; WT 28 dpi: 14.2 ± 1.32 ms, *n* = 12/4; KO 28 dpi: 13.3 ± 0.80 ms, *n* = 11/6 cells, *p* = 0.651, Mann–Whitney *U* test).

Previous studies have found that the decay kinetics of sIPSCs are slower in abDGCs than in mature granule cells because of the incorporation of α1 subunit into postsynaptic GABA_A_ receptors as neurons mature (Overstreet Wadiche et al., 2005). We tested whether the increased decay of the mIPSCs in abDGCs in *Fmr1* KO mice might reflect a lower α1 subunit incorporation by measuring the effect of zolpidem, an α1-specific positive allosteric modulator, on the decay kinetics of mIPSCs. mIPSCs were recorded at 21 dpi and the decay measured before and after the addition of 0.5 µM zolpidem (Fig. 2*I*). We found that zolpidem lengthened mIPSC decay in 21 dpi abDGCs in both *Fmr1* KO and WT to the same degree suggesting that α1 subunit content does not underlie the differences in decay observed in the *Fmr1* KO mice (decay zolpidem/decay control WT: 1.40 ± 0.11, *n* = 17/7; KO: 1.25 ± 0.05, *n* = 15/7, *p* = 0.87, Mann–Whitney *U* test).

### Maturation of the GABA reversal potential (*E*_GABA_) in abDGCs

**Figure 3.**
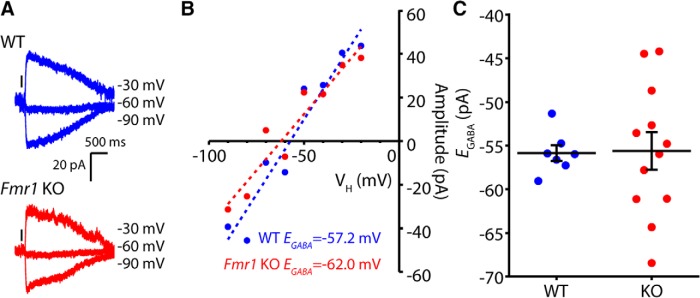
*E*_GABA_ in 21 dpi abDGCs in *Fmr1* KO and WT. ***A***, Representative traces of GABA responses from perforated-patch recordings in 21 dpi abDGCs in *Fmr1* WT (top, blue) and *Fmr1* KO (bottom, red) measured at three holding potentials (–30, –60, –90 mV). The response to a 50-ms puff of 10 μM GABA was measured at several holding potentials in the presence of 50 μM D-APV and 10 μM CNQX. ***B***, Representative current−voltage curves constructed from responses in two cells (blue WT, red KO). *E*_GABA_ was calculated as the *x*-axis intercept of the best-fit line of the current−voltage plot. ***C***, Grouped *E*_GABA_ data from all recordings.

As abDGCs mature the chloride reversal potential becomes progressively hyperpolarized in a similar manner to what occurs in other developing neurons (Ge et al., 2006; Chancey et al., 2013). The chloride equilibrium potential in large part determines the reversal potential for GABA_A_ receptors (*E*_GABA_) and therefore affects the strength of inhibitory transmission. *E*_GABA_ reaches its mature value 4 weeks after differentiation in abDGCs (Ge et al., 2006). Prior analysis of *Fmr1* KO mice has demonstrated that *E*_GABA_ maturation is delayed in the developing cortex and hippocampus of *Fmr1* KO mice (He et al., 2014; Tyzio et al., 2014). Therefore, we measured *E*_GABA_ using perforated patch recording from abDGCs 21 d after differentiation. In WT abDGCs the reversal potential was still depolarized from the mature value at this age (WT *E_GABA_*: –55.9 ± 0.91 mV, *n* = 7/3). However, this value did not differ from that recorded in abDGCs in *Fmr1* KO animals (KO *E_GABA_*: –55.6 ± 2.17 mV, *n* = 12/6, *p* = 0.967, Mann–Whitney *U* test; Fig. 3*A–C*). Therefore, while the reversal potential for GABA is still relatively immature and depolarized in 21 dpi abDGCs, there is no effect of the loss of FMRP on *E*_GABA,_ as has been reported in other developing neurons.

### Development of dendrites of abDGCs in *Fmr1* KO mice

**Figure 4.**
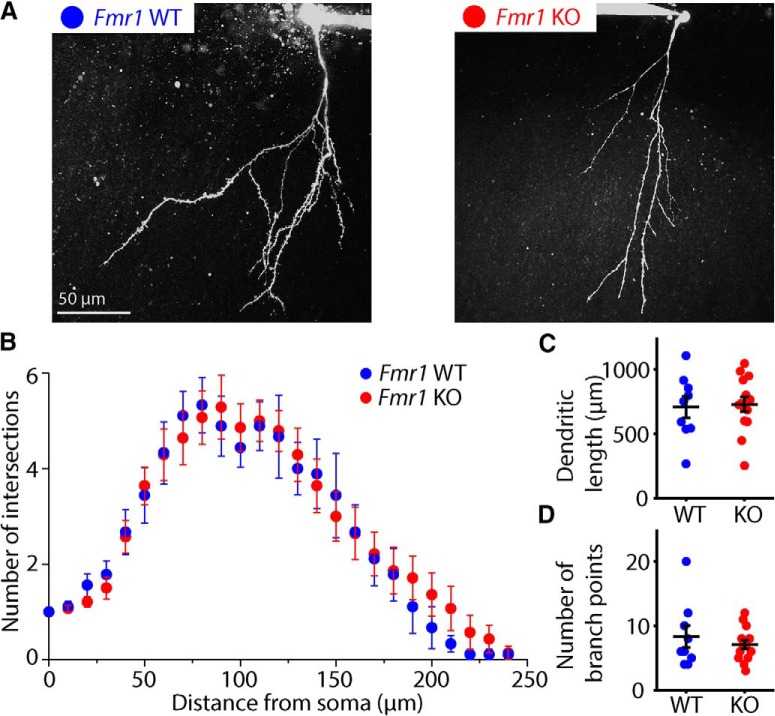
Morphology of 21 dpi abDGCs in *Fmr1* KO and WT. ***A***, Representative two-photon images of 21 dpi abDGCs filled with Alexa Fluor 488 in slices from *Fmr1* WT (right) and *Fmr1* KO (left) mice. ***B***, Sholl analysis of dendritic complexity of 21 dpi abDGCs in Fmr1 WT (blue) and Fmr1 KO mice (red). Average total dendritic length (***C***) and total number of branch points (***D***) of 21 dpi abDGCs in *Fmr1* WT (blue) and *Fmr1* KO (red) mice.

Dendritic morphology of abDGCs resembles that of mature DGCs as early as 21 dpi, when distal dendrites reach the outer molecular layer and significant arborization is observed (Zhao et al., 2006). Dendritic spines begin to form around 16 dpi, consistent with the fact that glutamatergic signaling is rarely observed before 14 dpi (Ge et al., 2006; Zhao et al., 2006). We thus sought to determine if loss of FMRP would lead to alterations in dendritic morphology in 21 dpi abDGCs during this critical period of their development. abDGCs were filled with a morphologic dye and imaged using a two-photon microscope (Fig. 4*A*). Quantification of their dendritic complexity at 21 dpi, as assessed by Sholl analysis did not uncover any significant difference between the genotypes (two-way ANOVA for unbalanced design, *p* = 0.999 for genotype × radius interaction; Fig. 4*B*). In addition, measurement of total dendritic length did not reveal any difference in 21 dpi abDGCs in *Fmr1* KO (WT: 701 ± 94.2 μm, *n* = 8/5; KO: 729 ± 57.5 μm, *n* = 15/8, *p* = 0.781, Mann–Whitney *U* test), and there was no difference in the number of dendritic branch points in *Fmr1* KO at 21 dpi (WT: 8.63 ± 1.92, *n* = 9/5, KO: 7.20 ± 0.66, *n* = 14/8, *p* = 0.917, Mann–Whitney *U* test; Fig. 4*C*,*D*).

## Discussion

In this study, we set out to systematically describe the development of abDGCs in the Fragile X mouse model, focusing on the formation of GABAergic inputs to these neurons during the first few weeks after differentiation. There are multiple studies that have found that loss of FMRP can delay neuronal development in the Fragile X brain, therefore, a similar delay in development of abDGCs could have an impact on how these neurons become connected to hippocampal circuits, and how they contribute to circuit function. Studying GABA synapse development is particularly relevant as these are the first synapses to form onto abDGCs and GABA also produces a trophic effect on abDGCs (Ge et al., 2006; Song et al., 2013; Alvarez et al., 2016). Surprisingly, our data indicate that the development of inhibitory signaling onto abDGCs during the first four weeks is mostly unaffected by the loss of FMRP in the *Fmr1* KO mice. Given the range of impairments in GABA signaling that have been described in the hippocampus of *Fmr1* KO mice including altered expression of GABA receptor subunits and GAD 65/67, and the alterations in *E*_GABA_ in *Fmr1* KO mice (Braat and Kooy, 2015a), it is surprising that GABA signaling appears to be unaltered in developing abDGCs during the critical period of their maturation.

### Critical period development and chloride homeostasis in Fragile X

At the cellular level Fragile X is a complex disorder because loss of FMRP leads to the dysregulation of expression of many neuronal proteins (Tang et al., 2015). The mouse model has been particularly useful in describing these complex alterations in neuronal development (Contractor et al., 2015). An important aspect that has emerged from some of these studies is the alterations in synaptic development of neurons during early critical periods in the cortex (Cruz-Martín et al., 2010; Harlow et al., 2010; Nomura et al., 2017). While these numerous delays in both excitatory and inhibitory neuron maturation have been well documented, up until now, it has not been established whether the development of abDGCs shows similar alteration in maturation to the developmentally generated neurons.

Among the known effects in developing neurons is the delay in the maturation of the GABA reversal potential in both the somatosensory cortex (He et al., 2014) and the hippocampus (Tyzio et al., 2014). In abDGCs, *E*_GABA_ has been demonstrated to be depolarized during early maturation in the weeks following differentiation (Tozuka et al., 2005; Ge et al., 2006) in a manner similar to developing neurons in other regions of the brain in perinatal mice. This occurs because of the relatively elevated intracellular chloride concentrations in abDGCs established by the juvenile chloride transporter NKCC1 (Ge et al., 2006). Disrupting chloride homeostasis in abDGCs leads to a profound alteration in the formation of synapses and in the maturation of the dendritic arbor. As there is growing evidence of altered *E*_GABA_ in developing neurons, we also measured *E*_GABA_ in abDGCs 21 d after differentiation when this measure is still maturing and not at its adult value (Ge et al., 2006). We confirmed that in 21 dpi abDGCS in both WT and *Fmr1* KO mice, the measured *E*_GABA_ is still relatively depolarized, but the values of *E*_GABA_ were not significantly different between the genotypes. Therefore, this crucial measure that regulates the strength of GABA signaling, and has a major impact on neuronal development, is not affected in abDGCs in *Fmr1* KO mice.

### GABA and Fragile X

The sequence of the formation of inputs and neurotransmitter signaling onto abDGCs broadly reflects that of developing neurons in other brain regions, including the early establishment of tonic and phasic GABAergic signaling followed by the establishment of excitatory connections (Ge et al., 2007a). GABA has an established role in brain development affecting proliferation (LoTurco et al., 1995; Haydar et al., 2000), migration, and maturation of progenitors and neurons (Represa and Ben-Ari, 2005; Wang and Kriegstein, 2009). After postnatal development, there are multiple known defects in GABA signaling associated with Fragile X, including age- and region-specific changes in GABA_A_ receptor subunit expression (Braat and Kooy, 2015b), changes in tonic and phasic GABA currents onto excitatory neurons (Centonze et al., 2008; Curia et al., 2009; Olmos-Serrano et al., 2010; Zhang et al., 2017) as well as defects in interneurons themselves (Gibson et al., 2008; Nomura et al., 2017). Expression of several GABA_A_ receptor subunits is reduced in the cortex, hippocampus, or forebrain of *Fmr1* KO mice (El Idrissi et al., 2005; D'Hulst et al., 2006; Gantois et al., 2006; Adusei et al., 2010). Expression of the glutamate decarboxylase enzyme (GAD) responsible for converting glutamate to GABA is increased in the hippocampus, but decreased in the amygdala of *Fmr1* KO mice (El Idrissi et al., 2005; Olmos-Serrano et al., 2010). Functionally, both the frequency and amplitude of mIPSCs and sIPSCs is reduced in the amygdala of juvenile *Fmr1* KO mice (Olmos-Serrano et al., 2010); however, a similar alteration is not observed in the subiculum of older mice (Curia et al., 2009). In some cases, these alterations in GABAergic signaling occur only early in postnatal development (Adusei et al., 2010; Nomura et al., 2017). Thus, loss of FMRP clearly affects GABA signaling in the postnatal mouse brain, but whether this is also the case for abDGCs was not known. By mapping spontaneous events by recording both sIPSCs and mIPSCs at three time points after differentiation of abDGCs, we were able to establish how inhibitory synapses form onto these neurons. We found that in both genotypes the frequency of IPSCs increases over time as would be expected if new synapses were being formed over the post differentiation period we analyzed. Interestingly, the amplitude of both the sIPSC and mIPSC events increased between 14 and 21 dpi. The increase in the mean quantal size at this developmental time point suggests that individual synapses become stronger. At the earliest time points, the amplitude of both mIPSCs and sIPSCs is equivalent suggesting that presynaptic axons of the inhibitory cells make a single contact whereas by 21 dpi the mean amplitude of sIPSCs is double the mean amplitude of quantal events, suggesting that the sIPSCs represent the release at multiple synapses. While again we did not observe any genotype differences in these parameters, we did observe consistent lengthening of the decay kinetics of mIPSCs in *Fmr1* KO mice which were significant in recordings from both 21 dpi and 28 dpi neurons. Prior work that compared the decay kinetics of inhibitory events in abDGCs and mature granule neurons found that the sIPSCs in abDGCs are slower because of the subunit composition of postsynaptic GABA_A_ receptors (Overstreet Wadiche et al., 2005). This study found that the zolpidem sensitivity of inhibitory events was increased in mature neurons suggesting that the α1 subunit is increasingly incorporated into neurons as they mature (Overstreet Wadiche et al., 2005). Given this, we considered the possibility that a reduction in the incorporation of the α1 subunit in abDGCs in *Fmr1* KO mice could underlie the prolonged decay of mIPSCs in *Fmr1* KO. However, we did not observe a significant difference in the effect of zolpidem on mIPSC decay in 21 dpi abDGCs between *Fmr1* KO and WT, indicating that a reduction in α1 expression is unlikely to underlie the changes in mIPSC decay. It is possible that alterations in expression of other GABA receptor subunits or even differences in the location of GABAergic synapses on developing neurons may underlie the observed change in mIPSC decays recorded by a somatic electrode. Interestingly, while prior work has reported that abDGCs lack expression of the GABA_A_ α1 subunit (Overstreet Wadiche et al., 2005), we found zolpidem had a significant effect on most mIPSCs in the 21 dpi birth-dated neurons in both genotypes. This may reflect differences in the populations of neurons that were analyzed in the previous study which recorded from POMC-GFP labeled neurons which are a more heterogeneous aged group (Overstreet et al., 2004).

In addition to the measures of synaptic function, we also measured the dendritic arbor to quantify both the complexity as well as the total dendritic length in 21 dpi abDGCs. A prior study has reported that selective deletion of FMRP in adult neural stem cells isolated from the dentate gyrus, as well as *in situ*, caused a reduction in both the dendritic complexity and total dendritic length when measured in neurons 56 d after differentiation (Guo et al., 2011). Dendritic length and complexity are also reduced in mice with knockout of the FMRP paralog FXR2P and double knockout of FXR2P and FMRP induced an additive effect on dendrites of abDGCs (Guo et al., 2015). In our experiments, we patched and filled abDGCs with morphologic dyes in live slices and imaged the dendritic arbor at 21 dpi. In these younger neurons, we did not observe any genotype related differences in the total dendritic length or complexity in *Fmr1* KO mice. It is possible that this difference in our results and those of the earlier study is due to a dendritic phenotype that emerges later on in the development of FMRP lacking abDGCs. While there have also been reports of region-specific or developmental age-specific alterations in dendritic spine density or immature spine morphology in *Fmr1* KO mice, there is no consensus on the effect of loss of FMRP on dendritic spines (for review, see He and Portera-Cailliau, 2013). While we did not examine this measure in these immature abDGCs, prior work has reported normal dendritic spines in the mature DG in *Fmr1* KO (Grossman et al., 2010).

In summary, we performed a systematic analysis of the functional properties of GABAergic synapses in abDGCs during the first four weeks after differentiation in *Fmr1* KO mice. While previous studies have demonstrated that cell proliferation and fate specification of adult neural stem cells is altered by FMRP loss, our data suggest that neurons that develop from these stem cells do not have major alterations in the maturation of their GABAergic synaptic inputs and dendrites during the first four weeks of their development.

## Synthesis

Reviewing Editor: Christian Hansel, University of Chicago Decisions are customarily a result of the Reviewing Editor and the peer reviewers coming together and discussing their recommendations until a consensus is reached. When revisions are invited, a fact-based synthesis statement explaining their decision and outlining what is needed to prepare a revision will be listed below. The following reviewer(s) agreed to reveal their identity: Molly Huntsman, Emanuela Santini.

The paper has the potential to advance the field, but more work needs to be done to increase its impact. The study shows that abDGC neurons in Fmr1 KO mice have an immature phenotype with respect to the lengthening of mIPSC decay during their early development (at 21dpi and 28dpi). This is possibly a consequence of their postsynaptic GABAa subunit composition. However, lack of FMRP does not change other inhibitory synaptic properties or the morphological phenotype of the abDGCs. This study suggests that the absence of FMRP does not affect the development of neurons derived from neuronal stem cells.

Statistics:

The Kolmorov-Smirnovtest may be the more appropriate statistical test here, as it is more sensitive to differences in distributions. Inclusion of the KS statistics and probability inter-event intervals and frequency may be warranted.

Comments

Overall the paper is well-written, the experiments well-designed and executed and the statistic is appropriate.

The impression is that the authors do not fully explain and exploit the difference in lengthening decay of abDGCs when Fmr1 is genetically ablated. So there is space to improvements-these are some suggestions:

In the discussion section of the paper, the authors speculate that this phenotype may be due to a reduction of the alpha1 subunit incorporation in abDGCs of Fmr1 KO mice.

The paper would be improved by performing an experiment with zolpiderm to show that this is indeed the case in Fmr1 KO mice. Editorial note: this experiment was strongly suggested by BOTH reviewers, and thus should have preference in the revision plans.

The authors should also expand the discussion of this point-for instance, it will be interesting to speculate on the reason(s) of the reduction of alpha1 subunit incorporation in relation to the known function of FMRP as inhibitor of protein synthesis (maybe the reduced incorporation of alpha1 subunit is caused by enhanced translation of the other subunits?)

The authors reference a paper (Overstreet Wadiche et al., 2005) where it is showed that sIPSCs kinetic decay in newborn neurons decay more slowly than in mature granule cells. The mIPSCs seem to have opposite trend in WT (fig2 F-G-H), do the authors have data on sIPSCs? if so, this should be included in the paper or at least, the authors should comment on this.

The graphs in Fig2 G and H should have the same scale.

Line 227. The authors recorded 7 neuron from 30 mice (n=7/30) !?!. Please, revise.

Fig 3. In panel B seems that WT and KO have different sensitivity to GABA although the Egaba is very similar. The authors should comment on this.

In panel A the representative traces seem to support this difference in sensitivity (very obvious at -90). The authors should find a more representative traces or comment on this.

The authors analyzed different morphological parameters of these neurons. It is known that a main morphological difference in neurons of FXS model mice (and patients) is the

difference in spine density and increase density of immature spines. Do the authors have data on the spine density of abDGCs? If not, they should at least comment on this.

The study aims to investigate the functional development of adult born Dentate Granule Cells (abDGCs) and the effect of loss of the fragile-x mental retardation protein (FMRP) on synaptic maturation and integration. To that end, the investigators identified abDGCs in Fmr1 by retroviral labeling in the subgranular zone and performed patch-clamp recordings to assess synaptic function of abDGCs across three time points post-injection. The investigators assess the frequency and peak amplitudes of spontaneous and mini IPSCs (sIPSCs, mIPSCs) and found no difference in inhibitory synaptic connections or strength between the groups. The decay kinetics, however, were found to be significantly increased in the KO animal. The investigators went on to evaluate the GABA reversal potentials and dendritic morphology in abDGCS of the wild-type and Fmr1 animal and, again, observed no differences.

The authors' findings imply that abDGCs in the Fmr1 mouse track with normal development in the wild-type animal. This study has the potential to extend the observations of GABAergic signaling in Fragile X Syndrome to other areas of the post-natal mouse brain. However, clarification and additional data are needed to reinforce the impact of this study - including more careful statistical treatment of data, and further studies. Major and minor comments are below.

MAJOR POINTS

It is reported that mean quantal size indicates increased strength. This study would be significantly strengthened by including experiments aimed at characterizing short and long-term potentiation in the dentate gyrus. Do abDGCs in the Fmr1 animal exhibit altered plasticity? Or is there a change in paired pulse inhibition?

The author's study deviates importantly from other studies in the literature regarding dendritic morphology changes and tonic and phasic inhibition in the Fmr1 KO animal. A more careful consideration of the literature and discussion about these interesting results is warranted.

Additionally, where there any significant changes in soma shape of abDGCs?

The authors state the earliest time point for observations of evoked inhibitory events reflected in the literature was 7 dpi. However, in their system, the earliest time point for spontaneous events is stated to be 14 dpi. To demonstrate this, a 7dpi time point for spontaneous events should be included in Figure 1. This may be a typo, but an evaluation of stimulus-evoked IPSCs may be warranted across these time points.

All Figures - scale and calibration bars should be clearly labeled on all images

MINOR POINTS

Manuscript should be reviewed for grammatical and syntactical errors.

Abbreviations should be defined at first appearance in manuscript (i.e the abbreviation “abDGCs” first appears in the abstract but is not defined until the significance statement.

For clarity, references to figures should be mentioned in the text in the order they presented in the figures.
